# Fractional exhaled nitric oxide in preterm‐born subjects: A systematic review and meta‐analysis

**DOI:** 10.1002/ppul.24270

**Published:** 2019-01-29

**Authors:** Christopher W. Course, Sailesh Kotecha, Sarah J. Kotecha

**Affiliations:** ^1^ Welsh Regional Neonatal Intensive Care Unit University Hospital of Wales Cardiff UK; ^2^ Department of Child Health School of Medicine Cardiff University Cardiff UK

**Keywords:** asthma, bronchopulmonary dysplasia, chronic lung disease of prematurity, FeNO, fractional exhaled nitric oxide, prematurity

## Abstract

**Background:**

Decreased lung function is common in preterm‐born survivors. Increased fractional exhaled nitric oxide (FeNO) appears to be a reliable test for eosinophillic airway inflammation especially in asthma. We, systematically, reviewed the literature to compare FeNO levels in preterm‐born children and adults who did or did not have chronic lung disease of prematurity (CLD) in infancy with term‐born controls.

**Methods:**

We searched eight databases up to February 2018. Studies comparing FeNO levels in preterm‐born subjects (<37 weeks’ gestation) in childhood and adulthood with and without (CLD) with term‐born subjects were identified and extracted by two reviewers. Data were analysed using Review Manager v5.3.

**Results:**

From 6042 article titles, 183 full articles were screened for inclusion. Nineteen studies met the inclusion criteria. Seventeen studies compared FeNO levels in preterm‐ and term‐born children and adults; 11 studies (preterm *n* = 640 and term *n* = 4005) were included in a meta‐analysis. The mean FeNO concentration difference between the preterm‐born and term‐born group was −0.74 (95% CI −1.88 to 0.41) ppb. For the six studies reporting data on CLD (preterm *n* = 204 and term *n* = 211) the mean difference for FeNO levels was −2.82 (95% CI −5.87 to 0.22) ppb between the preterm‐born CLD and term‐born groups.

**Conclusions:**

Our data suggest that preterm born children with and without CLD have similar FeNO levels to term‐born children suggesting an alternative mechanism to eosinophilic inflammation for symptoms of wheezing and airway obstruction observed in preterm‐born subjects.

## 1 INTRODUCTION

Adverse events in early life and their impact on longer term health and morbidity are increasingly recognized.^1^ Preterm birth is a well‐recognized early adverse event, with potential lifelong health implications. Approximately 11% of all infants are born prematurely world‐wide with rates increasing in many countries.^2^ Prediction and prevention of preterm birth is currently limited thus characterizing the long‐term effects of preterm birth is vital to optimize their future quality of life.

The lungs are one of the last major body system to mature in utero, therefore, preterm infants are born with immature airway structures with decreased gas exchange surface.^3^ Respiratory pathology is the commonest consequence of preterm birth, manifesting as neonatal respiratory distress syndrome, a consequence of structurally immature lungs and pulmonary surfactant deficiency. These infants often require ventilatory support, both invasive and non‐invasive, and supplementary oxygen. A subset of these infants will go on to develop chronic lung disease of prematurity (CLD),^4^ often defined as supplementary oxygen requirement at 36 weeks’ corrected gestational age.^5^

Nitric oxide (NO) has several functions in the lung including neurotransmission, vasodilatation, bronchial dilatation, and immune enhancement. However, in diseases such as asthma, its actions can be paradoxical. Animal studies have shown that at lower concentrations, NO has a bronchodilator effect, but at higher levels NO has a pro‐inflammatory action. The increased levels of NO observed in disease states caused by airway inflammation are thought to be the result of increased induction of nitric oxide synthease and of increased reactive nitrosylation pathways.^6^

Fractional exhaled nitic oxide (FeNO) has been demonstrated to be a useful biomarker of eosinophilic airway inflammation. FeNO levels can be easily measured non‐invasively by a single‐breath exhalation technique and, through utilizing mathematical airway modelling, is thought to provide a representation of distal airway inflammation.^6^ Although commonly thought of as an eosinophilic disease, airway inflammation in asthma can also be neutrophilic in origin.^7^ FeNO levels have been shown to be higher in children with asthma and during asthma exacerbations, being associated with increased airway inflammation.^8^ It was reported in a systematic review in 2017 that FeNO is a reasonably accurate diagnostic test for identifying subjects with asthma, with good specificity and acceptable sensitivity,^9^ especially in those with eosinophilic lung disease. FeNO is not only widely used as a diagnostic tool, but serial measurements have also been shown to be useful for monitoring treatment responses to inhaled corticosteroids.^10^

Several studies have shown evidence of persistent abnormal lung function in later childhood for infants diagnosed with CLD. In previous systematic reviews, we have reported that preterm‐born subjects have deficits in FEV_1_, increased rates of bronchial hyper‐responsiveness, and single doses of bronchodilators appeared to improve FEV_1_.^11^, ^12^, ^13^ It has been reported that preterm‐born subjects have higher rates of wheezing and are often diagnosed with asthma. The systematic review by Been et al. reported increased rates of wheezing in preterm‐born subjects.^14^ In addition, we have reported increased rates of wheezing in preterm‐born children which was independent of a family history of atopy.^15^ We would question the diagnostic labelling of preterm‐born subjects with the label asthma, especially as the mechanisms underlying the deficits in lung function and increased rates of bronchial hyper‐responsiveness remain uncertain. We believe that the mechanisms are likely to differ to asthma thus sought if FeNO was a useful diagnostic test for preterm‐born children as it is for asthma.

Thus, we conducted a systematic review and meta‐analysis to identify:

- if FeNO level is increased after preterm birth when compared to term‐born controls, and
- if FeNO level is increased in preterm‐born subjects who had CLD compared to term‐born subject.

## 2 METHODS

We ran search strategies which included keywords relating to FeNO, preterm‐birth and study design in eight databases; and additionally we searched references in the included articles to identify additional papers reporting FeNO levels in preterm‐born subjects compared to term‐born subjects (see Supplementary Protocol for protocol, search strategy, and data collection table). The eight databases searched were Web of Knowledge, Scopus, CINAHL, OpenSIGLE, Medline, Medline in process, EMBASE, and HMIC. Ethical approval was deemed not necessary.

### 2.1 Eligibility criteria

Studies on FeNO levels in preterm‐born children and adults subjects over 2 years of age, with or without CLD compared to term‐born subjects were included. Preterm‐born was defined as birth at <37 weeks’ gestation and term‐born as birth at ≥37 weeks’ gestation. We accepted the authors’ definitions of CLD. All methods of assessing FeNO levels were accepted. However, only studies which reported the concentration of FeNO in the preterm‐born and a comparator term‐born groups were included. Studies in all languages from all countries were considered.

### 2.2 Study selection

Searches were carried out in February 2018. Two reviewers (SJK and CC) independently screened each reference title and available abstracts, using the inclusion criteria in the protocol. Any reference title that met the inclusion criteria as judged by either CC or SJK was then included in the next stage where the complete full manuscript was obtained. SJK and CC then reviewed the full manuscript against the inclusion criteria. Where there was agreement, the manuscript was included in the next stage and data were extracted. Where there was disagreement, a third reviewer (SK) reviewed the complete manuscript and arbitrated the final decision.

### 2.3 Data collection process

SJK data extracted data from included articles into the data tables, which were independently verified by CC. If data was not in a format which enabled the data to be included in the systematic review, the authors were contacted for further information wherever possible. Where multiple manuscripts from the same cohort were identified, they were reviewed by SJK and SK and a decision made about which article should be included, based on the largest, most complete cohort.

### 2.4 Outcome measures

FeNO level in the preterm‐born subjects and in the preterm‐born subjects with CLD compared with a term control group.

### 2.5 Analysis of results

A formal meta‐analysis was conducted for the studies which reported the mean and standard deviation, (or where they could be easily obtained) of FeNO (a) in the preterm (with and without CLD) and control groups, and (b) in the preterm with CLD group and term control group. The results of all the studies are also presented descriptively.

### 2.6 Statistical analysis

Statistical analyses were performed using Review Manager (RevMan) Version 5.3.^16^ After initial exploration of the data, we used random effects meta‐analyses to allow for heterogeneity. Medians were converted to means using the method of Wan et al. where enough information was present to allow this to be calculated.^17^ A calculator was used to convert 95% confidence intervals to standard deviation.^18^ In addition, a sensitivity analysis was conducted: studies where the means and standards deviations were calculated from medians, and the single study where standard deviations were calculated from 95% confidence intervals were removed from the meta‐analysis.

## 3 RESULTS

### 3.1 Studies selected and their characteristics

**Figure 1:**
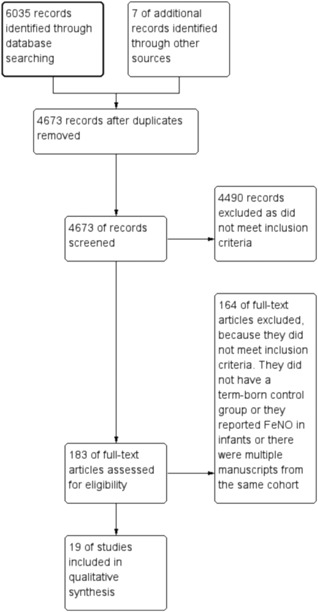
Study selection results

A total of 6042 article titles were identified of which 183 full articles were screened for inclusion. Nineteen met the inclusion criteria (Figure 1).^19^, ^20^, ^21^, ^22^, ^23^, ^24^, ^25^, ^26^, ^27^, ^28^, ^29^, ^30^, ^31^, ^32^, ^33^, ^34^, ^35^, ^36^, ^37^ For the three studies that overlapped data were kindly supplied by the authors.^32^, ^33^, ^34^ In addition, a further author kindly supplied their data so the study could be included.^35^ Demographics of included articles are shown in Supplementary Table S1. Medians and IQRs or ranges were converted to means and standard deviations for three studies.^23^, ^25^, ^28^ Mean and 95% CI were converted to mean and standard deviation for one study.^24^

### 3.2 Study outcomes

#### 3.2.1 FeNO in preterm group compared to term control group

**Figure 2:**
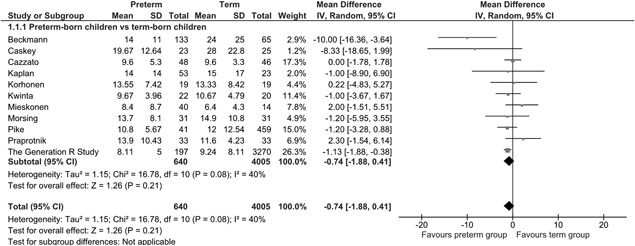
Mean FeNO of the preterm‐born group compared to the term‐born group

From the 17 studies identified, 11 (preterm *n* = 640 and term *n* = 4005) were included in a meta‐analysis and 6 are described in detail below. The results of the meta‐analysis are shown in Figure 2. The mean difference for FeNO level between the preterm‐born and term‐born group was −0.74 (95% CI −1.88 to 0.41) ppb. The studies are described in detail in Supplementary Table S1.

#### 3.2.2 FeNO in preterm group who had CLD in infancy compared to term control group

**Figure 3:**
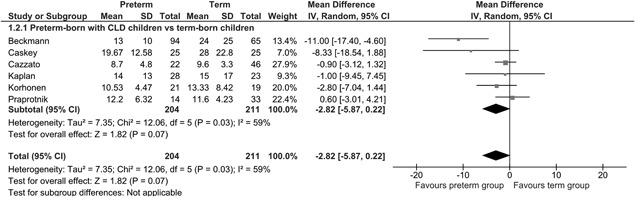
Mean FeNO of the preterm‐born group with CLD compared to the term‐born group

From the 17 studies identified, 6 (preterm *n* = 204 and term *n* = 211) were included in a meta‐analysis as shown in Figure 3. The mean difference for FeNO levels between the preterm‐born and term‐born group was −2.82 (95% CI −5.87 to 0.22) ppb. The studies are described in detail in Supplementary Table S1.

#### 3.2.3 Sensitivity analysis

Removing the four studies where the means and/or standard deviations were calculated from the meta‐analysis reporting the FeNO levels in the preterm group compared to the term group did not alter the mean difference substantially (mean −0.92 (95% CI −2.28 to 0.45) ppb. Removing the three studies where the means and/or standard deviations were calculated from the meta‐analysis reporting the FeNO levels in the preterm group who had CLD in infancy compared to the term group did alter the mean difference substantially (−4.11 (mean 95% CI −10.72 to 2.50) ppb. However, by removing the three studies, only three studies were left in the meta‐analysis and the number of preterm‐born children dropped from 204 to 144, with the number of term‐born children dropping from 211 to 134.

#### 3.2.4 Description of studies not included in the meta‐analyses

Six studies were not included in a meta‐analysis. They are described in detail in Supplementary Table S1 and briefly summarised below with data for FeNO given in ppb. In general, the preterm‐born subjects with and without CLD compared to the term‐born group had statistically similar or lower levels of FeNO compared to control groups. Most were excluded as they either reported data as geometric means or as medians.

1. Nordlund et al.^19^ studied 30 preterm‐born children with CLD and 30 term born children with asthma sensitized to airborne allergens at a mean age of approximately 10 years and reported a significantly lower FeNO level in the preterm‐born children with CLD (median FeNO in the CLD group 7.9, in the asthma group 13.3).
2. Baraldi et al.^20^ studied 31 preterm‐born children with CLD, 31 preterm‐born children without CLD, 31 term controls and 31 patients with asthma at an approximate mean age of 8 years. The two preterm‐born groups had the lowest FeNO levels when compared to the term controls and the patients with asthma. The CLD group had statistically significantly lower levels of FeNO compared to the other three groups (geometric mean FeNO in the CLD group 7.7, term 10.7, preterm‐born no CLD 9.9, asthma 24.9).
3. Kilbride at al.^21^ studied extremely low birth‐weight preterm‐born children, heavier preterms and term‐born children with a normal birthweight at 12‐15 years of age FeNO results did not differ by birthweight category (FeNO in ELBW preterm‐born children 10.2, heavier preterm born children 15.2, and normal birthweight term‐born children 10.4).
4. Vollsaeter at al.^30^ also studied 45 preterm‐born adults, who were subdivided by CLD severity, and compared the CLD groups to 39 term‐born, 18‐25 year‐old adults. FeNO levels did not differ between the groups (geometric mean FeNO terms 11.8, No‐CLD 11.7, Mild CLD 12.2, moderate/severe CLD 11.5).
5. Vollsaeter at al.^36^ studied preterm‐born children with and without CLD and term‐born controls at a mean age of approximately 11 years. FeNO levels did not differ significantly between the groups (geometric mean FeNO terms 11.77, preterm no CLD 10.46, preterm CLD 9.08).
6. Malmberg et al.^37^ studied very low birthweight children, children with a history of wheeze and term‐born children at 8‐10 years of age. The FeNO levels were not significantly different between the groups once height was adjusted for (geometric mean FeNO terms 9.9, and VLBW 10.7).

## 4 DISCUSSION

To our knowledge, this is the first systematic review and meta‐analysis examining FeNO levels in preterm infants, with and without CLD, compared to infants born at term. FeNO level is generally higher in subjects with asthma, but especially in those who have eosinophilic airway inflammation. FeNO has been established as a reliable, non‐invasive test for aiding the diagnosis of eosinophilic asthma, with reasonable sensitivity and good specificity.^9^ A recent review studying exhaled biomarkers summarized the evidence to date with the statement that “FeNO is consequently considered a marker of a common asthma endotype characterized by Th2‐mediated airway inflammation, eosinophilia, and responsiveness to inhaled steroids”.^8^ The results of our systematic review and meta‐analyses suggest that subjects born preterm have no statistically significant difference in FeNO levels when compared to term‐born subjects. Preterm‐born subjects with CLD also had similar results to term‐born subjects. The latter results should be treated with caution as the number of studies and total number of subjects was smaller; and the mean difference was altered by the removal of the studies where the means and/or standard deviations were calculated from medians or 95% CIs.

Despite improvements in neonatal management over the past 20 years, including the routine use of antenatal steroids, surfactant, and gentle mechanical ventilation modes, CLD remains a significant consequence of preterm birth at an immature stage of pulmonary development. Preterm born infants are known to experience long‐term respiratory morbidity during the rest of childhood. Those with a diagnosis of CLD are most vulnerable. Preterm born infants with and without CLD are more prone to wheezing symptoms, more frequently use inhaled bronchodilator treatments, are more likely to be admitted to hospital for management of wheeze or dyspnoea, and are more than twice as likely to be labelled with a diagnosis of asthma.^38^ This increased respiratory morbidity appears to extend beyond childhood into adulthood.^39^ However, the reasons for continuing respiratory illness in childhood and beyond remains unclear.

Our results suggest that preterm‐born subjects are unlikely to have eosinophilic airway inflammation underlying their respiratory symptoms and may not have the same airway mechanics as those children with a diagnosis of asthma. The data suggest that there may be alternative underlying mechanisms to explain their episodic or chronic wheezing and symptoms of airway obstruction. Whether that is structural or functional remains to be confirmed; our recent systematic review reported increased bronchial hyper‐responsiveness in preterm‐born subjects compared to term‐born subjects by both direct and indirect methods of measuring bronchial hyper‐responsiveness. When only subjects who were preterm‐born and had CLD in infancy were studied, the differences were greater, suggesting greater pathology of their airways.^13^ We have also reported increased exercise‐induced bronchial hyper‐responsiveness in school‐age children with a background of CLD which is responsive to bronchodilator therapy.^40^

With increasing number of children surviving preterm‐birth, it is important to understand the impact of preterm birth at a critical stage of lung development. Currently, the mechanisms underlying the increased respiratory symptoms, deficits in lung function, increased bronchial hyper‐responsiveness, responses to single doses of a bronchodilator are not fully understood. Our data adds that airway eosinophilic inflammation is unlikely since FeNO level was not increased in the preterm groups including in those with CLD when compared to their term counter‐parts. However, FeNO may be affected by abnormal lung development in patients with CLD, so it will be difficult to exclude completely eosinophilic airway inflammation. We have recently shown that preterm‐born subjects may have specific wheezing phenotypes.^41^ It may be that specific phenotypes are associated with increased FeNO. There are suggestions that neutrophilic or oxidant injury may be continuing but these small studies need to be replicated including to identify the reasons why such injury is continuing especially given that the initial injuries most likely occurred perinatally.

The authors used various different methods to measure FeNO and, importantly, used differing methods to represent their data including as means, medians or geometric means; or as proportions of children with greater than a predefined value of FeNO, most commonly 35 ppb. This led to difficulty in interpreting the data between the different studies. Thus, it is important in any future task force where expert opinion is sought to include the most appropriate way of reporting FeNO concentrations. Given that the data is unlikely to be normally distributed, geometric means together with proportions of children with greater than predefined FeNO values may be the most appropriate way forward.

## 5 LIMITATIONS

As with all systematic reviews we were limited by the quality and data supplied in the studies included in the meta‐analysis. The data was not always normally distributed hence many authors did not present their results as means and standard deviations. We converted medians where possible to means and standard deviations, but this may have introduced errors. However, we carried out a sensitivity analysis to explore this. In addition, the heterogeneity of the studies included is also a limitation. There was heterogeneity due to the fact the preterm and term‐born subjects were born over a number of years, were tested at a range of ages, and were born at a range of gestations, during a period where standards of neonatal intensive care and management of extreme prematurity have changed, including the introduction of antenatal steroids and surfactant. In addition, FeNO levels were not measured with same make of device in all the included studies.

## 6 CONCLUSION

Our systematic review and meta‐analysis showed that infants born preterm, both with and without CLD had similar FeNO values to term‐born controls. Despite preterm born children being more likely to show wheezing symptoms and be labelled with a diagnosis of asthma, our data suggests that their respiratory symptoms and airway obstruction are unlikely to be mainly due to eosinophilic airway inflammation thus unlikely to benefit from the diagnostic label of asthma. Future research should aim to further the underlying mechanisms explaining the long‐term respiratory morbidity observed in preterm‐born children and to accurately identify biomarkers to tailor their treatments to their specific respiratory pathology.

## CONFLICT OF INTEREST

The authors have no conflicts of interest to report.

## Supporting information

Additional supporting information may be found online in the Supporting Information section at the end of the article.

Supporting Data S1.

Supporting Table S1.
