# Supplementary material for: Fractional exhaled nitric oxide in preterm‐born subjects: A systematic review and meta‐analysis
Source: Pediatr Pulmonol. 2019 Jan 29;54(5):595–601. doi: 10.1002/ppul.24270 (PMC6519366; doi:10.1002/ppul.24270)
Supplement: Supplementary file 1 — Supporting Data S1. [file PPUL-54-595-s001.docx]

**SUPPLEMENTARY PROTOCOL**

**Fractional exhaled nitric oxide in preterm-born subjects: A systematic review and meta-analysis.**

^2^Chris Course BSc MBBCh MRCPCH, ^1^Sailesh Kotecha PhD FRCPCH, ^1^Sarah J Kotecha PhD.

^1^Department of Child Health, School of Medicine, Cardiff University, Cardiff, UK.

^2^Welsh Regional Neonatal Intensive Care Unit, University Hospital of Wales, Cardiff, UK

**Protocol**

FeNO in preterm-born subjects compared to term born subjects - a systematic review of the literature.

Review Question

Is there increased FeNO in preterm-born subjects compared to subjects born at term?

Outcome of interest

FeNO results

Study population

Studies that include people of any age living in any country. Studies published from any year.

Search strategy

A search strategy was developed for electronic databases using the keywords and MeSH headings below. The search strategy was tested for citations on the OVID Medline database 1950-2010. The Observational Studies search filter used by SIGN (Scottish Intercollegiate Guidelines Network) <http://www.sign.ac.uk/methodology/filters.html#obs> was adapted to retrieve types of study designs included in the review.

The search strategy will be modified to search rest of the bibliographic databases. In addition, a range of ‘snowballing’ techniques will be used to increase the sensitivity of the search, including reference list follow up, contact with subject experts and relevant websites/organisations, and table of content scanning for the top three most frequently cited journals.

Inclusion and exclusion process of identified citations

The agree search strategy will be applied to the agreed databases and information sources.

A single Reference Manager file will be produced of all references identified through the search process. Duplicates will be removed. Each reference will be given a unique identifier code number.

Two electronic copies of references in this file will be produced, and one set given to each reviewer. Two people will review the papers.

Both reviewers will independently screen each reference title and abstract (if available) from their copies the file, using the agreed inclusion and exclusion criteria. Reviewers will receive an electronic spreadsheet to indicate yes for probable or possible inclusion for each citation, or no for exclusion. At this stage abstracts only will be translated if the citation is not written in English.

The independent screening assessments will be merged.

Where both reviewers have indicated no for a citation then that citation will be excluded at this stage, and the full article will not be obtained.

Where both reviewers have indicated yes for a citation then the full article will be obtained. They will go into the second round of inclusion/exclusion assessment of articles.

Where one reviewer has indicated yes and the other no then the full article will be obtained and go into the second round as above.

The second round will involve each reviewer assessing the full article based on the agreed inclusion/exclusion criteria. Each will decide yes or no for exclusion for each citation reaching the second round.

The second round assessments will be merged.

Where both indicate yes in the second stage the citation will go forward for subsequent data extraction and critical appraisal. Where both indicate no the citations will be excluded. Where there is disagreement then a third reviewer will apply the inclusion/exclusion criteria and their yes or no will be final.

Inclusion criteria

Studies clearly or potentially primarily about

- Subjects born prematurely compared to subjects born at term and containing FeNO data should be included.

Other criteria

- The study population can be of any age and sex.
- The study population can be from any country.
- The studies must be randomised or non randomised intervention studies; prospective or retrospective cohort studies; or prospective or retrospective case control studies.
- Studies written in any language.

Exclusion Criteria

- Case reports, case series, letters, editorials or expert opinions only.
- Studies primarily about the clinical management of broncho pulmonary dysplasia or other lung conditions or management of the lung when the premature infant is born.

Languages included

All languages will be considered.

Study data extraction and critical quality appraisal

Data extraction form.

Study designs included

1. Randomised and non-randomised intervention studies.
2. Prospective and retrospective case control studies.
3. Prospective and retrospective cohort studies

Study characteristics to be collected

1. Relevance or appropriateness of studies gathered for assessing hypothesis to be tested.
2. Rationale for the selection and coding of data.
3. Documentation of how data were classified and coded.
4. Assessment of confounding.
5. Assessment of study quality.
6. Assessment of heterogeneity.
7. Statistical methods.

Results

Descriptive information

Descriptive information for each included study. Details of study design, participants, interventions, definitions of outcomes, will be included in the table of characteristics of included studies. Between study heterogeneity will be assessed and explained. The reasons for excluding any studies will be clearly reported.

Search strategy

Ovid MEDLINE – Search Strategy

| 1 | exp Infant, Low Birth Weight/ | 30718 |
| --- | --- | --- |
| 2 | exp Infant, Very Low Birth Weight/ | 8894 |
| 3 | exp Infant, Extremely Low Birth Weight/ | 1562 |
| 4 | exp Infant, Premature/ | 49475 |
| 5 | Obstetric Labor, Premature/ | 12835 |
| 6 | exp Premature Birth/ | 10472 |
| 7 | Fetal Growth Retardation/ | 14772 |
| 8 | Birth Weight/ | 36538 |
| 9 | Low Birth Weight.mp. | 34566 |
| 10 | ((Preterm* or Premature*) adj2 (labo#r* or birth* or born or infant or baby or babies or child or children or girl*1 or boy*1)).mp. | 83061 |
| 11 | Prematurity.mp. | 18289 |
| 12 | IUGR.mp. | 4475 |
| 13 | intrauterine growth restriction.mp. | 4293 |
| 14 | or/1-13 | 154572 |
| 15 | Epidemiologic Studies/ | 7569 |
| 16 | exp case control studies/ | 890992 |
| 17 | exp cohort studies/ | 1702215 |
| 18 | Case control.tw. | 92757 |
| 19 | (cohort adj (study or studies)).tw. | 124284 |
| 20 | Cohort analy*.tw. | 5103 |
| 21 | (Follow up adj (study or studies)).tw. | 41098 |
| 22 | (observational adj (study or studies)).tw. | 63466 |
| 23 | Longitudinal.tw. | 167485 |
| 24 | Retrospective.tw. | 350481 |
| 25 | Cross sectional.tw. | 219200 |
| 26 | Cross-sectional studies/ | 255166 |
| 27 | case-control studies/ or longitudinal/ or follow-up studies/ or prospective studies/ | 1183258 |
| 28 | or/15-27 | 2333933 |
| 29 | exp Nitric Oxide/ | 81967 |
| 30 | FeNO.mp. | 1296 |
| 31 | exp Breath Tests/ | 13578 |
| 32 | exp Exhalation/ | 3316 |
| 33 | exp Nitric Oxide/ | 81967 |
| 34 | exhaled nitric oxide.mp. | 2984 |
| 35 | nitric oxide.mp. | 145607 |
| 36 | sildenfil.mp. | 3 |
| 37 | or/29-36 | 158826 |
| 38 | 14 and 28 | 44708 |
| 39 | 37 and 38 | 227 |

The following table is an explanation of the symbols used in the search strategy above.

| / | after an index term (MeSH heading) indicates that all subheadings were selected. |
| --- | --- |
| * | before an index term indicates that that term was focused - i.e. limited to records where the term was a major MeSH/Emtree term. |
| "exp" | before an index term indicates that the term was exploded. |
| .tw. | indicates a search for a term in title/abstract |
| .mp. | indicates a free text search for a term |
| # | retrieves records that contain the search term with substituted character(s) in the specified location. |
| * | at the end of a term indicates that this term has been truncated. |
| *n | The limited truncation symbol, $n, Retrieves records that contain the search term and all possible suffix variations of a root word with the maximum number of characters that may follow the root word or phrase, specified by n. |
| ? | in the middle of a term indicates the use of a wildcard. |
| adj | indicates a search for two terms where they appear adjacent to one another |

Databases and information sources

| Bibliographic databases |
| --- |
| CINAHL 1982- |
| Embase 1980- |
| [HMIC Health Management Information Consortium](http://ovidsp.tx.ovid.com/sp-2.3/ovidweb.cgi?New+Database=Single\|7&S=NOGIFPIBLHDDEIBJMCELIFNKKMNPAA00) 1979 |
| Medline 1950- |
| Medline in Process |
| Scopus |
| OpenSIGLE |
| Web of Knowledge  Science Citation Index Expanded 1981-  Social Science Citation Index 1981-  ISI Proceedings 1990- |
