# Supplementary material for: Fractional exhaled nitric oxide in preterm‐born subjects: A systematic review and meta‐analysis
Source: Pediatr Pulmonol. 2019 Jan 29;54(5):595–601. doi: 10.1002/ppul.24270 (PMC6519366; doi:10.1002/ppul.24270)
Supplement: Supplementary file 2 — Supporting Table S1. [file PPUL-54-595-s002.docx]

**Table S-1 Demographics of included articles.**

**Fractional exhaled nitric oxide in preterm-born subjects: A systematic review and meta-analysis.**

**^2^Chris Course BSc MBBCh MRCPCH, ^1^Sailesh Kotecha PhD FRCPCH, ^1^Sarah J Kotecha PhD.**

**^1^Department of Child Health, School of Medicine, Cardiff University, Cardiff, UK.**

**^2^Welsh Regional Neonatal Intensive Care Unit, University Hospital of Wales, Cardiff, UK**

| **Study and COUNTRY** | | **Objective** | | **Study design** | | **Study Group** | | | | **Control Group** | | **Outcome Measures** | | | **Subjects (gender)** |
| --- | --- | --- | --- | --- | --- | --- | --- | --- | --- | --- | --- | --- | --- | --- | --- |
| Nordlund^1^  Sweden | | “The objective of this study was  to describe hallmarks of BPD at school age in comparison to children with atopic  asthma.” | | “cross-sectional descriptive comparative study” | | 30 BPD  13 mild  11 moderate  6 severe | | | “30 age- and sex-matched children with asthma and sensitized to  airborne allergens (IgE >0.35 kUA/L)” | | | FENO | | | BPD 20/30 67%  Asthma 19/30 (63%) |
| **GA (weeks)** | **BW (grams)** | | **Duration on mechanical ventilation (DAYS)** | **Age tested (years)** | | | **Year of birth** | **Surfactant given** | | | **Method of measuring** | | | **RESULTS** | |
| BPD 26.6  weeks’ gestation  Mild mean 27.1 SD 1.6,  Moderate/severe mean 26.1 SD 2.6  Asthma group born at term. | BPD  Mild mean 1128g SD 293,  Moderate/severe mean 924 SD 343 | | Assisted ventilation BPD  Mild median 5 IQR 8,  Moderate/severe median 14  IQR 14 | BPD average age 10.4 years SD 1.0  Asthma average age 10.7 years  SD 0.7 | | | 1998-1999 | BPD  Mild 82%,  Moderate/severe 63% | | | “FENO was measured in parts per billion (ppb) using NIOX® equipment (Aerocrine AB, Solna, Sweden) in accordance with guidelines.” | | | “FENO values were significantly lower in children with BPD (12 vs 23, P = 0.019).”  BPD median 7.9 IQR 8.9  Asthma median 13.3 IQR 25.3 | |
| **Study and COUNTRY** | | **Objective** | | | **Study design** | **Study Group** | | | | **Control Group** | | **Outcome Measures** | | | **Subjects (gender)** |
| Baraldi^2^  Italy | | “The aim of this study was to evaluate FeNO and lung function indices at school age in a cohort of survivors of BPD, comparing them with a group of matched healthy children and a group of preterm children without BPD. As control subjects with a similar degree of airflow limitation, we also included a group of children with asthma matched for airway function.” | | | Cross-sectional | 31 BPD  31 preterm children without BPD matched for birthweight and gestational age | | | Matched control subjects born at term, matched for sex and age.  31 children with asthma | | | FENO | | | Males/females  BPD 14/17.  Asthma 14/17.  Preterm 14/17.  Term 14/17. |
| **GA (weeks)** | **BW (grams)** | | **Duration on mechanical ventilation (DAYS)** | | **Age tested (years)** | | **Year of birth** | **Surfactant given** | | | **Method of measuring** | | **RESULTS** | | |
| BPD <31 weeks  Mean 28.6 SEM 0.3  Preterm mean 28.9 SEM 0.4 | BPD <2000g  Mean 1081 SEM 57  Preterm mean 965 SEM 40 | | BPD needed mechanical ventilation 7 days after birth.  Preterm without BPD did not require mechanical ventilation beyond the 7^th^ day of life  BPD Mean 26d SEM 3.4  Preterm mean 2.6d SEM 0.7 | | BPD mean 8.6 yr SEM 0.3.  Asthma mean 8.6 yr SEM 0.5.  Preterm BPD mean 8.7 yr SEM 0.3.  Term mean 8.4 yr SEM 0.4. | | 1990-1994 | BPD 24/31  Preterm 7/31 | | | “FeNO was measured with an online method using a computerized system  (EBA Aerocrine, Stockholm, Sweden) following the American Thoracic Society’s Recommendations (18). Subjects inhaled NO-free air  through the mouth to total lung capacity and exhaled through a dynamic flow restrictor with a target flow of 50 ml/second for at least 6–7 seconds). No nose clip was used. FeNO, expressed as ppb, was calculated as the mean of three measurements that agreed to within 10% of the mean value.” | | “Their FENO values (geometric mean [95% confidence interval]: 7.7 [+/- 1.1] ppb) were significantly lower than in a group of healthy matched control subjects born at term (10.7 [+/- 1.1] ppb, p < 0.05) and a group of preterm children without BPD (9.9 [+/- 1.1] ppb, p < 0.05). The children with BPD were also compared with a group of 31 patients with asthma with a comparable airflow limitation (FEV1 80.2 +/-2.1% predicted) and showed FENO values four times lower than in those with asthma (24.9 [+/-1.2] ppb, p<0.001).” | | |

| **Study and COUNTRY** | | **Objective** | | **Study design** | **Study Group** | | | | **Control Group** | | **Outcome Measures** | | **Subjects (gender)** |
| --- | --- | --- | --- | --- | --- | --- | --- | --- | --- | --- | --- | --- | --- |
| Kilbride^3^ | | “To assess the impact of preterm birth on childhood pulmonary function and oxygen consumption measurements. Secondary objective is to assess levels of exhaled nitric oxide in preterm versus term children and relationship to clinical findings.” | | Cross sectional | 12 extremely low birth weight (ELBW), 18 heavier preterms (HPT). | | | 9 born at term with normal birth weight (NBW). | | | FENO | | Not stated |
| **GA (weeks)** | **BW (grams)** | | **Duration on mechanical ventilation (DAYS)** | **Age tested (years)** | | **Year of birth** | **Surfactant given** | | | **Method of measuring** | | **RESULTS** | |
| Mean GA (ELBW 24.7+/-1.3 wk;  HPT 33.2+/-1.7;  NBW 38.9+/- 0.2). | Mean BW  (ELBW 674+/-83 g,; HPT 1952+/-278, NBW 3661+/-770). | | Not stated | 12-15 years | | 1993-1995 | Not stated | | | “Exhaled nitric oxide (FE,NO) levels were determined prior to exercise testing by utilizing NIOX Flex or NIOX MINO.” | | “33 children had FENO measurements, which were significantly higher in males (15.5+/-6.3 and 10.7+/-7.1 ppb, P=.038) but not different by BW category (10.2+/-9, 15.2+/-6, 10.4+/-6, P=.11). 3 children, all former preterms, had high FENO (>20 ppb); 8 other preterm children had intermediate FENO (16-20 ppb); however, FENO did not correlate with VO2max or PFTs.” | |

| **Study and COUNTRY** | **Objective** | | | **Study design** | **Study Group** | | | | **Control Group** | | **Outcome Measures** | | **Subjects (gender)** |
| --- | --- | --- | --- | --- | --- | --- | --- | --- | --- | --- | --- | --- | --- |
| Morsing^4^  Sweden | “To assess lung function at early school age in children delivered at very early gestation owing to intrauterine growth restriction and abnormal foetal blood flow (IUGR).” | | | prospective controlled study | 31 children born preterm with IUGR  Control groups  were matched for gender and age and had BW appropriate for gestational age (AGA); 31  children born preterm | | | 31 children born at term | | | FENO | | The study population 31 children (16 girls and 15 boys).  Other groups matched for gender. |
| **GA (weeks)** | | **BW (grams)** | **Duration on mechanical ventilation (DAYS)** | **Age tested (years)** | | **Year of birth** | **Surfactant given** | | | **Method of measuring** | | **RESULTS** | |
| (PT-IUGR)  median range) gestational age 26.9 (24–29) mean (SD) 27.0 (1.4) weeks.  (PT-AGA) g matched for gestational  age at birth, and mean (SD) 27.0 (1.5)  (T-AGA) born at term mean (SD)  39.9 (0.6) | | (PT-IUGR) with a median (range) birth weight (BW) of 650 (395–976) g.  (T-AGA) with BW of 3530 (3000–4390) g.  (PT-AGA) with BW of 1010 (660–1790) g matched for gestational  age at birth. | Not stated | median age 8.4 (range 6.5–10.7) years  PT-IUGR mean 8.4 SD (1.2)  PT-AGA mean 8.4 SD (1.3)  T-AGA mean 8.4 SD (1.3) | | 1998 and 2004 | Not stated | | | “Fractional exhaled nitric oxide (NO) was measured to  assess airway inflammation using the NIOX Mino (Aerocrine,  Stockholm, Sweden). The NO measurements took  place before the spirometric measurements” | | “Exhaled NO did not differ between the groups.”  Exhaled NO, PT-IUGR mean (SD) 11.0 (5.7),  PT-AGA 13.7 (8.1),  T-AGA 14.9 (10.8) | |

| **Study and COUNTRY** | | **Objective** | | **Study design** | **Study Group** | | | | **Control Group** | | **Outcome Measures** | | **Subjects (gender)** |
| --- | --- | --- | --- | --- | --- | --- | --- | --- | --- | --- | --- | --- | --- |
| Kwinta^5^  Poland | | “To determine if ELBW infants are at higher risk for the development of allergic and respiratory symptoms and to establish if there were any specific risk factors for these symptoms.” | | A cross-sectional observational study | 81 children born ELBW born with a gestational age <30 weeks and birth weight <1000 g  22 had FENO results. | | | The control group included 40 full-term children.  20 had FENO results | | | FENO | | ELBW Female 52/81 (64%)  Term 21/40 (53%) |
| **GA (weeks)** | **BW (grams)** | | **Duration on mechanical ventilation (DAYS)** | **Age tested (years)** | | **Year of birth** | **Surfactant given** | | | **Method of measuring** | | **RESULTS** | |
| ELBW mean gestational age of 27.2 weeks (SD: 2.1  weeks).  Control full term children.  Mean (SD) 39.9 (1.4) | ELBW <1000g born with a mean birthweight of 845 g SD 130g  Term Birth weight (g), mean (SD) 3554 (512) | | Not stated | 6-7 years of age. ELBW mean age 6.7 years. SD 0.4 | | 1 of September 2002 to the 31 of August 2004 | Not stated | | | “Exhaled nitric oxide (FeNO) was measured in concordance  with published standards [12], using an electrochemical  hand-held device - NIOX MINOW (Airway Inflammation Monitor /NIOX MINO/, Aerocrine AB, Solna, Sweden), following the producers instructions, with exhaled air flows equal to 50±5 ml/s. Measurements of FeNO were performed prior to all other study procedures. FeNO results were evaluated according to the guidelines by Taylor et al. in which - levels equal or below  20 ppb were regarded as normal” | | “All of the ELBW had normal FeNO level (<=20 ppb), but 5 children from the control group had abnormal results (p=0.02).” “Appropriate maneuvers for FeNO measurements were obtained in 22 ELBW children and in 20 of the controls.  All of the ELBW participants had normal FeNO levels (≤20 ppb). Five children from the control group had abnormal results: 3 had results ranging from 20–35 ppb and 2 above 35 ppb.”  FeNO (ppb)  Median (25th-75th percentile) ELBW 8 (8–13) Term 10 (8–14) p value 0.2  Normal (≤20 ppb) ELBW 22 term 15 p value 0.02  Intermediate (20–35 ppb) ELBW 0 term 3  High (>35 ppb) ELBW 0 term 2  N=81 N=40 | |
| **Study and COUNTRY** | | **Objective** | | **Study design** | **Study Group** | | | | **Control Group** | | **Outcome Measures** | | **Subjects (gender)** |
| Praprotnik^6^  Slovenia | | “To examine respiratory morbidity and exercise capacity at school age in children after bronchopulmonary  dysplasia (BPD).” | | Follow up study | Preterm children after BPD (n = 23) 14 had FENO, without BPD (n = 33) all had FENO | | | Term controls (n = 33)  All had FENO | | | FENO | | M:F  BPD 15:8  NON BPD 13:20  All preterms 28:28 terms 13:20 |
| **GA (weeks)** | **BW (grams)** | | **Duration on mechanical ventilation (DAYS)** | **Age tested (years)** | | **Year of birth** | **Surfactant given** | | | **Method of measuring** | | **RESULTS** | |
| Preterms 24 to 30 completed weeks  of gestation.  Mean (SD) 26.7 (1.7)  BPD (26.2 ± 2.4  NON BPD 27.2 ± 1.7 weeks);  Terms gestational age >37 weeks | Terms > 2800 g,  Preterms mean (SD) 996 (246)  BPD (867 ± 276 g)  Non BPD 1056 ± 252 g) | | “All of the 23  preterm BPD children had been intubated after birth, whereas 8  out of 33 children were intubated in the non-BPD group.”  “The  length of ventilatory support BPD (32 [95% confidence interval 18.0 to  49.0] days vs Non BPD 5.5 [3.5 to 21.5] days.” | 7-9 years  Preterms  BPD 7.5±1.0 NON BPD 7.7± 0.8  Preterms 7.6±0.9 Terms 8.0±1.0 | | 1 September 2000 and  30 June 2002. | “Surfactant treatment was administered to 86% of the children in  the group with prior BPD and to 9% in the non-BPD group” | | | “The fractional exhaled nitric oxide (FeNO) was measured in accordance with the recommendations of the ATS and the ERS. We used the single breath measurement  with a hand-held device (NioxMino, Aerocrine, Sweden) for at least 6 s. The FeNO measurements were performed by a single technician blinded to the clinical details of the participant. They were performed only in children without signs and symptoms of acute respiratory infection during the last 6 weeks. Children were asked not to eat or drink at least 1 h before the measurement. Because of the flow dependency of the FeNO values we encouraged the use of constant expiratory flow rates by using a visual computer program. Nose clips were not used. Three recordings were made, and the results in agreement within 10% were included in the  analysis.” | | “There were no differences in FeNO between BPD and the former preterm non- BPD or the full-term children. Children born preterm as a  group had statistically significant higher values of FeNO, however,  these differences were clinically insignificant.”  FeNO, ppb  BPD mean 12.2 95% CI 9.1 to 16.4  Non BPD mean 13.9 95% CI 10.7 to 18.1  Terms mean 11.6 95% CI 10.2 to 13.2 | |

| **Study and COUNTRY** | | **Objective** | | **Study design** | **Study Group** | | | | **Control Group** | | | **Outcome Measures** | | | **Subjects (gender)** |
| --- | --- | --- | --- | --- | --- | --- | --- | --- | --- | --- | --- | --- | --- | --- | --- |
| Korhonen^7^  Finland | | “Airway inflammation is involved in the pathogenesis of BPD. The aim of the study was to evaluate the inflammatory activity in plasma and exhaled air in BPD survivors at school age. | | Cross sectional | 21 VLBW (birth weight ≤1,500 g) children with severe radiographic BPD (radBPD), 19 without  radBPD (nonBPD group). | | | 19 non-asthmatic term controls | | | | FeNO | | | radBPD 14 (67%)  nonBPD 11 (58%) |
| **GA (weeks)** | **BW (grams)** | | **Duration on mechanical ventilation (DAYS)** | **Age tested (years)** | | **Year of birth** | **Surfactant given** | | | **Method of measuring** | | | | **RESULTS** | |
| Term mean (SD) gestational age was  39.5 (1.7) weeks.  radBPD, 26.6 (1.6) nonBPD 28.9 (1.8) | Term mean (SD) birth weight 3,499  (610) g.  radBPD 919 (252)  nonBPD 1,198 (222) | | radBPD 19 (91%)  Duration of ventilator therapy (d), MD (range) 24 (0–271)  nonradBPD 11 (58%)  Duration of ventilator therapy (d), MD (range) 1 (0–5) | 6-14 years | | January 1st, 1995,  and April 13th, 2003 | radBPD 14 (67%)  nonBPD 9 (47%) | | | “NO concentrations in exhaled air were measured with a Sievers NOA 280 analyzer (Sievers Instruments, Boulder, CO) at four exhalation flow rates (50, 100, 200, and 300 ml/sec), as previously described.18 NO output (product of eNO concentration and exhalation flow rate) was calculated and plotted against the exhalation  flow rate. A regression line was set to correlate these variables, alveolar NO concentration and bronchial NO flux constituting the slope and intercept of the regression line, respectively. The alveolar NO concentration  reflects NO dynamics in the peripheral lung (from respiratory bronchioles to alveoli), and bronchial NO flux the NO dynamics in central conducting airways. | | | | “There were no significant differences between the groups in any of the inflammatory markers measured.”  “Exhaled breath FENO50 (ppb) radBPD MD (range) 8.2 (4.40–21.30), n=16  nonBPD 8.7 (4.70–32.1), n=18  Term 7.1 (4.0–35.1), n=18  Also reported alveolar and Bronchial FENO. | |
| **Study and COUNTRY** | | **Objective** | | **Study design** | **Study Group** | | | | **Control Group** | | | **Outcome Measures** | | | **Subjects (gender)** |
| Beckmann^8^  UK | | “The aim was to evaluate respiratory outcomes in young adults born at ≤25 weeks gestation.” | | Cohort | 124  EP subjects | | | 64 term-born controls | | | | FeNO | | | Not stated abstract |
| **GA (weeks)** | **BW (grams)** | | **Duration on mechanical ventilation (DAYS)** | **Age tested (years)** | | **Year of birth** | **Surfactant given** | | | | **Method of measuring** | | **RESULTS** | | |
| EP ≤25 weeks gestation  Controls term | Not stated abstract | | Not stated abstract | 19 years. | | Not stated abstract | Not stated abstract | | | | Not stated abstract | | “FeNO levels were lower in the EP group, with no correlation with FEV1.”  FeNP ppb +/- SD  EP + BPD (n=94) 13 (10)  EP no BPD (n=37) 15 (13)  All EP 14 (n=133) (11)  All control (n=65) 24 (25)  Ep to control p<0.01 | | |

| **Study and COUNTRY** | | **Objective** | | **Study design** | **Study Group** | | | | **Control Group** | | **Outcome Measures** | | **Subjects (gender)** |
| --- | --- | --- | --- | --- | --- | --- | --- | --- | --- | --- | --- | --- | --- |
| Cazzato^9^  Italy | | “The aim of this study was to assess pulmonary function and its predictors in VLBW ≤ 1,500 g) children (VLBWc) with or without BPD, born at gestational age ≤32 weeks at a single tertiary center  during 1996–1999, after the introduction of surfactant therapy” | | Cross sectional | 48 (40%) VLBWc 26 children  who did not develop BPD (no-BPD subgroup) and 22 children with prior BPD (BPD subgroup) | | | 46 age matched  controls (BW > 2,500 g and  GA > 37 weeks) | | | FeNO | | Male/female  No BPD 15/11  BPD 12/10  VLBW 27/21  Controls 25/21 |
| **GA (weeks)** | **BW (grams)** | | **Duration on mechanical ventilation (DAYS)** | **Age tested (years)** | | **Year of birth** | **Surfactant given** | | | **Method of measuring** | | **RESULTS** | |
| No BPD 30 (28–32)  BPD 26 (24–30)  VLBW 28 (24–32)  Control  39 (37–40) | No BPD 1,220g ( 230)  BPD 840g (160) VLBW 1,050g ( 278)  Control  3,150g (567) | | Days of MV—median (IQR)  No BPD 0 (0)  BPD 2 (11)  VLBW 0 (3) | No BPD 8.4 (1.2)  BPD 8.7 (0.8)  VLBWc 8.5 +/- 1.0 yearS  controls 8.8+/- 1.4 years | | 1996-1999 | No BPD 20%  BPD 94%  VLBW 56% | | | **“**Exhaled FeNO was measured using a chemiluminescence  analyzer with an on-line method using a computerized  system (Ecomedics; Durnten, Switzerland) following the ATS-ERS recommendations.” | | **“**No differences were found in FeNO levels between VLBWc and controls.”  FeNO (ppb)  No BPD 10 (5.6)  BPD 8.7 (4.8)  VLBW 9.6 (5.3)  Controls 9.6 (3.3) | |

| **Study and COUNTRY** | | **Objective** | | **Study design** | **Study Group** | | | | **Control Group** | | **Outcome Measures** | | **Subjects (gender)** |
| --- | --- | --- | --- | --- | --- | --- | --- | --- | --- | --- | --- | --- | --- |
| Caskey^10^  N Ireland | | “To assess structural and functional lung parameters in young adult BPD survivors  and preterm and term controls” | | Cohort | 25 adult BPD survivors,  24 adult prematurely born non-BPD  Both preterm groups  were (VLBW: < 1500g). | | | 25 adult term birth controls | | | FeNO | | Gender, male  BPD 14/25 (56%) Non BPD 14/24 (58%)  Control 15/25 (60%) |
| **GA (weeks)** | **BW (grams)** | | **Duration on mechanical ventilation (DAYS)** | **Age tested (years)** | | **Year of birth** | **Surfactant given** | | | **Method of measuring** | | **RESULTS** | |
| BPD survivors, (mean ± SD)  26.8 ± 2.3 weeks range 23-30;  non-BPD controls  30.6 ± 1.9 weeks range 26-34;  controls (GA 38.5 ± 0.9) | BPD survivors, (mean ± SD)  866 ± 255 g) range 510-1490,  non-BPD controls  (1234 ± 207 g) range 760 to 1500,  controls (3569 ± 2979 g) | | BPD Duration of IPPV, h 966 (599-1652) | BPD (mean (SD) age 24.0 (3.4)y, range 19 to 33y).  non-BPD controls (mean (SD) age 26.4 (3.7)y, range 21 to 34y)  term controls (mean (SD) age 28.3(3.3)y, range 22 to 33y). | | January 1978 and April  1993. | BPD 8/25 (32%)  Non BPD 0/24 (0%)  Control 0/25 (0%) | | | “FeNO was measured using the (NIOXTM MINO (Aerocrine AB,  Sweden) according to ATS/ERS recommendations.” | | **“**Although FeNO measurements were lower in both BPD subjects (median 19, IQR: 12 to 28 ppb) and non-BPD controls (median 19, IQR: 12 to 28 ppb) compared to term controls (median 27, IQR: 14 to 43 ppb) neither result attained statistical significance.” | |

| **Study and COUNTRY** | | **Objective** | | **Study design** | **Study Group** | | | | **Control Group** | | **Outcome Measures** | | **Subjects (gender)** |
| --- | --- | --- | --- | --- | --- | --- | --- | --- | --- | --- | --- | --- | --- |
| Kaplan^11^  Israel | | “To determine the long-term pulmonary outcome of extreme prematurity at a single tertiary care centre” | | cohort | 28 BPD 25 without BPD | | | 23 Terms | | | FeNO | | All PT 26M 49.1% BPD 15M 53.6%, No BPD 11M 44% Controls 13M 56.5% |
| **GA (weeks)** | **BW (grams)** | | **Duration on mechanical ventilation (DAYS)** | **Age tested (years)** | | **Year of birth** | **Surfactant given** | | | **Method of measuring** | | **RESULTS** | |
| All PT born ≤28 weeks mean (SD) 26.7 weeks +/- 1.3  BPD 26.2+/- 1.4 weeks;  No BPD 27.2 +/- 1 weeks  23 term control subjects ≥37 week 39.8 +/- 1.38. | All PT mean (SD) 930 +/-205  BPD 821 +/- 164 g  No BPD, 1,050+/- 181 g  Term were all ≥ 2500g 3230 +/- 413 | | PT median 4 range 0-48 BPD 13 (0-48) non BPD 2 (0-9) | ALL PT mean (SD) 10+/- 1.5 years  All PT 10 +/- 1.5 BPD 9.7 +/- 1.7, No BPD 10.3 +/- 1.4 Controls 9.9 +/-1.2 | | 1997-2001 | 44/53 83% PT received  postnatal surfactant.  BPD 24/28 85.7%, non BPD 20/25 80% | | | **“**Feno (normal, <20 parts per billion [ppb]) was measured using  a chemiluminescence analyzer (CLD 77 AM; ECO MEDICS  AG). The sample rate was 100 Hz, and flow sampling rate was  200 mL/min by American Thoracic Society/European Respiratory  Society recommendations. | | FeNO ppb mean (SD)  All PT 14 (14)  BPD 14 (13)  Non BPD 14 (15)  Term 15 (17)  NS difference | |

| **Study and COUNTRY** | | **Objective** | | **Study design** | **Study Group** | | | | **Control Group** | | **Outcome Measures** | | **Subjects (gender)** |
| --- | --- | --- | --- | --- | --- | --- | --- | --- | --- | --- | --- | --- | --- |
| Vollsaeter^12^  Norway | | “To compare changes from 18–25 years of age in respiratory health, lung function, and airway responsiveness in young  adults born extremely prematurely to that of term-born control subjects.” | | Population based sample | 45 Preterm (gestational age ≤28 wk or birth weight ≤1,000 g)  Non-BPD n = 11  Mild BPD n=22  M/S BPD n = 12 | | | 39 matched term controls | | | FENO | | EP 19/45 (42%) were females  Control 18/39 (46%) were females |
| **GA (weeks)** | **BW (grams)** | | **Duration on mechanical ventilation (DAYS)** | **Age tested (years)** | | **Year of birth** | **Surfactant given** | | | **Method of measuring** | | **RESULTS** | |
| EP mean (range)  27.4 (1.5) (23–32) weeks,  ).  Non-BPD 28.5 (1.6) Mild BPD 27.0 (1.3)  M/S BPD 27.2 (1.5)  Control term | EP mean (range)  1,006 (193) (580–  1480) g  Non-BPD 1,151 (155) Mild BPD 1,001 (197)  M/S BPD 883 (123)  Control,  mean (SD) birth weight was 3,445 (299) g, | | Median (range), d  Non-BPD 0.3 (0–4.8)  Mild BPD 8.3 (0.8–35)  M/S BPD 25.4 (0.7–54) | 25 years | | 1982-1985 | Not stated | | | **“**Fractional exhaled  nitric oxide (FENO) was measured with  Exhalyzer CLD-88 (EcoMedics, Durnden,  Switzerland) according to ATS/ERS standards” | | “FENO single breath (P = 0.949)  and FENO nasal (P = 0.777) did not differ between the two groups, with no influence from neonatal BPD.”  FENO 0.05 sa,geometric mean Control 11.8 (9.3–14.9)  All EP 11.8 (9.5–14.7)  Non-BPD 11.7 (7.7–17.9)  Mild BPD 12.2 (9.0–16.6)  M/S BPD 11.5 (7.7–17.2)  FENO na, ppb All control 939.9 (794.6–1,085.2)  EP 947.0 (803.5–1,090.5)  Non-BPD 911.9 (627.4–1,196.4)  Mild BPD 825.4 (629.9–1,021.0)  M/S BPD 1,103.6 (846.3–1,361.0) | |

| **Study and COUNTRY** | | **Objective** | | **Study design** | | **Study Group** | | | **Control Group** | | **Outcome Measures** | | **Subjects (gender)** |
| --- | --- | --- | --- | --- | --- | --- | --- | --- | --- | --- | --- | --- | --- |
| Mieskonen^13^  Finland | | “To investigate the inflammatory basis of abnormal pulmonary function in CLD, we measured eNO in school-age children who had been born very prematurely,  with or without CLD, and we compared the results with the values of healthy, nonatopic controls born at term.” | | Follow up study | | 40 children born  very prematurely with gestational  ages of ≤30 weeks or birth weights of<1,500 g | | 14 nonatopic term-born controls | | | FENO | | PT Boys 19 (48%) |
| **GA (weeks)** | **BW (grams)** | | **Duration on mechanical ventilation (DAYS)** | **Age tested (years)** | **Year of birth** | | **Surfactant given** | | | **Method of measuring** | | **RESULTS** | |
| Gestational age at birth, weeks 27.9 (24.1–30.9) | PT birth weights, Birth weight, g 990 (600–1,575) | | “PT all except one child required mechanical ventilation after  birth. Ventilator treatment, days 10 (0–75)” | PT 7.5–9.6 years, years median (range) 8.3 (7.5–9.6).  Control 8.9 (5.3–11.2) | 1989–1991, | | Not stated | | | “Exhaled nitric oxide was measured using a chemiluminescence  analyzer (Sievers 270B, Boulder, CO) with a rapid-response time (<200 msec) and an accuracy of _1 ppb. In addition, the analyzer also measured expiratory flow and exhaled volume in real time. The sampling rate through the reaction chamber of the analyser was 250 mL/min for all measurements. The analyzer was calibrated daily, using NO-free oxygen to set the absolute zero and a certified NO gas of 184 ppb NO in nitrogen (AGA Edelgas, Germany).For eNO measurements, after inhaling NO-free oxygen, the children exhaled slowly from total lung capacity for 15 sec through a flow resistor (Hans Rudolph, Model  7100R, 50 cmH2O/L/sec, flow range 0–0.5 L/sec), which created a positive pressure necessary to close the soft palate. Due to the small lung volumes of the children, the target flows recommended by the European Respiratory  Society (ERS)27 (0.16–0.25 L/sec) appeared to be unsuitable, as they required a volume of at least 2.4–3.7 L for an exhalation of 15 sec. Therefore, lower target flows were used in this study (0.10–0.20 L/sec). A visual computer program was used to help to maintain a fairly  constant flow. A nose clip was used, and cheeks and lips were supported to prevent any gas leak. During a steady exhalation, eNO reaches a plateau after 5–10 sec, while exhaled volume continues to increase. The mean value of  the last 3 sec of the end-expiratory plateau of NO concentration  was taken for the analysis. Results of analyses were computed and graphically displayed on a plot showing eNO levels, flow, and volume against time, and expressed as eNO concentration (ppb) and NO output (pmol/sec). At least 3 successive recordings were made,  with a coefficient of variation of less than 10%. The mean value of measurements was recorded in the case record forms..” | | **“**In children born prematurely, eNO was significantly higher in atopics than in nonatopics (respective means, 14.8 vs. 6.3 ppb, P=0.02). Nonatopic prematurely born infants did not differ significantly from controls (means, 6.3 vs. 6.4 ppb, P=ns).” In nonatopic schoolchildren born very prematurely and with a history of CLD, we found no evidence of airway inflammation associated with increased eNO concentrations. Neither were  eNO levels associated with severity of chronic lung disease, as determined by conventional  lung function tests. eNO levels were higher in atopic children born prematurely than in controls. In the group of children born prematurely, the mean (SD) exhaled NO level was 8.4 (8.7) ppb, which did not differ significantly from the 6.4 (4.3) ppb of the controls. The NO output was 44.8 (41.2) pmol/sec and 30.8 (24.8) pmol/sec, respectively (P=ns). In the prematurely born group, atopic children (N¼10) had significantly higher eNO values than nonatopic children (N=29) (mean (SD) 14.8 (14.2) ppb vs. 6.3 (4.5) ppb, P=0.02). The NO output values of atopic and nonatopic children were 68.7 (56.6) and 36.8 (33.8) pmol/sec, respectively; this was a significant difference (P=0.04). The eNO concentrations and NO output  values of nonatopic children born prematurely did not differ significantly from those of controls. The mean (SD) eNO concentration  was 6.8 (4.6) ppb in the CLD group, 5.9 (4.8) ppb in the non-CLD group, and 5.9 (4.9) ppb in the control  group, with no significant differences between groups. NO output values were 48.7 (47.9), 29.5 (23.6) and 30.8 (24.8) pmol/sec, respectively, with no significant differences between groups” | |

| **Study and COUNTRY** | | **Objective** | | **Study design** | **Study Group** | | | | **Control Group** | | **Outcome Measures** | | **Subjects (gender)** |
| --- | --- | --- | --- | --- | --- | --- | --- | --- | --- | --- | --- | --- | --- |
| The Generation R Study^14-16^  The Netherlands  (Data supplied by authors) | | Data from authors | | Population based prospective cohort | 197 preterms <37 weeks gestation | | | 3270 terms ≥37 weeks gestation | | | FeNO | | Not given data supplied by authors. |
| **GA (weeks)** | **BW (grams)** | | **Duration on mechanical ventilation (DAYS)** | **Age tested (years)** | | **Year of birth** | **Surfactant given** | | | **Method of measuring** | | **RESULTS** | |
| preterms <37 weeks gestation.  Terms ≥37 weeks gestation | Not given data supplied by authors. | | Not given data supplied by authors. | 6 years old | | Not stated | Not given data supplied by authors. | | | Fractional exhaled nitric oxide (FeNO), a noninvasive  biomarker of eosinophilic airway inflammation,  was measured in parts per billion (ppb) using the NIOX chemiluminescence analyser (Aerocrine AB,  Solna, Sweden). | | PT mean (SD) 8.11 (5.00)  Terms mean (SD) 9.24 (8.11) | |

| **Study and COUNTRY** | | **Objective** | | **Study design** | **Study Group** | | | | **Control Group** | | **Outcome Measures** | | **Subjects (gender)** |
| --- | --- | --- | --- | --- | --- | --- | --- | --- | --- | --- | --- | --- | --- |
| Pike^17^  UK | | Data from authors | | Human mother-child cohort | 41 preterms | | | 459 terms | | | FeNO | | Not given data supplied by authors. |
| **GA (weeks)** | **BW (grams)** | | **Duration on mechanical ventilation (DAYS)** | **Age tested (years)** | | **Year of birth** | **Surfactant given** | | | **Method of measuring** | | **RESULTS** | |
| Not given data supplied by authors | Not given data supplied by authors | | Not given data supplied by authors | 6 years of age | | Test between 2006-2010 | Not given data supplied by authors | | | “eNO Was measured according to European Respiratory Society  /ATS recommendations using a NIOX chemiluminescence analyser (Aerocrine, Sweden). A mean of three readings was calculated where possible. Values were normalised by inverse square root transformation then standardised. The sign was reversed so that high standardised scores represent high untransformed eNO values.” | | Data kindly supplied by authors  Terms mean 11.998003 SD 12.54104  preterms mean 10.799593 SD 5.6733948  . | |

| **Study and COUNTRY** | **Objective** | | | **Study design** | | **Study Group** | | | **Control Group** | **Outcome Measures** | | **Subjects (gender)** |
| --- | --- | --- | --- | --- | --- | --- | --- | --- | --- | --- | --- | --- |
| Vollsaeter^18^  Norway | “Compare respiratory health in children born extremely preterm (EP) or with extremely low birthweight (ELBW) nearly one decade apart, hypothesizing that better perinatal management has led to better outcome.” | | | Population based controlled longitudinal studies | | 57 children <28 weeks gestation or <1000g  Non BPD 26 BPD 31  Fe_NO_ was successfully measured in 49/57 EP, with alveolar NO obtained from 45/57, respectively, missing data mainly due to equipment failure the first months of the study. | | | 54 Matched term controls >37 weeks birthweight >3000g  Fe_NO_ was successfully measured in 48/54 term-born subjects, with alveolar NO obtained from 43/54, missing data mainly due to equipment failure the first months of the study. | Exhaled FeNO | | Control 25 F  PT 28 F  Non BPD 16 F BPD 12 F |
| **GA (weeks)** | | **BW (grams)** | **Duration on mechanical ventilation (DAYS)** | **Age tested (years)** | **Year of birth** | | **Surfactant given** | **Method of measuring** | | | **RESULTS** | |
| Control >/=37 weeks  PT 26.8 SD 1.6  Non BPD 27.3 (1.6)  BPD 26.3 (1.4) | | Control mean 3701 SD (434)  PT 850 (175)  Non BPD 873 (200)  BPD 831 (151) | PT Median 5.0 range (0-24)  Non BPD 2.5 (0-21)  BPD 8.0 (0-24) | Control mean 11.7 SD (0.7)  PT 11.4 (0.6)  Non BPD 11.4 (0.6)  BPD 11.5 (0.6) | 1999-2000 | | PT 49/56  Non BPD 20/25  BPD 29 | “Fractional exhaled nitric oxide (FeNO) was measured with Exhalyzer CLD-88 (EcoMedics, Switzerland), according to ATS/ERS recommendations).” | | | FeNO 0.05 G. mean  Controls 11.77 (9.53,14.54)  All EP 9.65 (7.97,11.69)  EP non BPD 10.46 (7.59,14.43)  EP BPD 9.08 (7.08,11.65)  Mean difference (95% CI) All EP vs. Control -1.22 (-1.62, 1.09)  * P values EP vs Control 0.18  FeNO did not differ significantly between the EP-born and term born. | |
| **Study and COUNTRY** | **Objective** | | | **Study design** | | **Study Group** | | | **Control Group** | **Outcome Measures** | | **Subjects (gender)** |
| Malmberg^19^  Finland | “To investigate the association between AHR and fractional exhaled nitric oxide (FeNO) including the alveolar concentration of nitric oxide, in school-age children with VLBW” | | | Cohort | | 29 children with VLBW (</= 1500g), 12 with BPD 28 with successful lung function tests, 33 children with a history of early wheeze | | | 60 healthy controls | FeNO | | VLBW male 13/28  Wheeze 22/33  Control 30/60 |
| **GA (weeks)** | | **BW (grams)** | **Duration on mechanical ventilation (DAYS)** | **Age tested (years)** | **Year of birth** | | **Surfactant given** | **Method of measuring** | | | **RESULTS** | |
| Control and wheeze group were term. VLBW mean (range) 28 (25-31) | | VLBW mean (range) 991 (520-1453) | VLBW mean (range) 15d (0-52) | 8 to 10 years  VLBW mean (range) 9.7 (9.1-10.3)  Wheeze 8.2 (7.6-9.0)  Control 8.6 (8.1-9.3) | January 1995 to August 1996 | | VLBW 16/28 | “FeNO was measured using computerised equipment with a chemiluminescence analyser according to ERS/ATS recommendations. The system was calibrated with a certified nitric oxide calibration gas mixture according to the manufacturers’ instructions. Exhalations that did not meet the ATS requirements were rejected by the system, and the child was asked to perform new exhalation maneuvers, until 3 reproducible FeNO values within 10% wew obtained. The mean FeNO was recorded.”. | | | “Geometric mean FeNO levels were similar between VLBW children and healthy controls, and a history of BPD had no effect.”  “ The geometric means (IQR) of FeNO levels between the VLBW group (mean 10.7 ppb; IQR 7.6-12.5 ppb) and healthy controls (mean 9.9 ppb; IQR 7.6-12.1 ppb) were similar (p=.96). The history of BPD had no effect Compared with controls the children born full term with a history of wheeze had slightly lower FeNO levelS with a geometric mean of 8.9ppb (IQR 6.1-7.4 PPB), but after adjusting for the height difference between the groups, the levels were statistically similar (p=.14).” | |

References

1. Nordlund B, James A, Ebersjo C, Hedlin G, Brostrom EB. Differences and similarities between bronchopulmonary dysplasia and asthma in schoolchildren. Pediatric Pulmonology 2017;52(9):1179-1186.

2. Baraldi E, Bonetto G, Zacchello F, Filippone M. Low exhaled nitric oxide in school-age children with bronchopulmonary dysplasia and airflow limitation. American Journal of Respiratory and Critical Care Medicine 2005;171(1):68-72.

3. Kilbride H, Dinakar C, Carver T, Gauldin C, Teson K, Gelatt M, Sabath R. Pulmonary function, oxygen consumption, and exhaled nitric oxide measures for extremely low birth weight, heavier preterm, and term children. Journal of Investigative Medicine 2012;60 (1):239.

4. Morsing E, Gustafsson P, Brodszki J. Lung function in children born after foetal growth restriction and very preterm birth. Acta Paediatrica, International Journal of Paediatrics 2012;101(1):48-54.

5. Kwinta P, Lis G, Klimek M, Grudzien A, Tomasik T, Poplawska K, Pietrzyk JJ. The prevalence and risk factors of allergic and respiratory symptoms in a regional cohort of extremely low birth weight children (< 1000 g). Italian Journal of Pediatrics 2013;39.

6. Praprotnik M, Gantar IS, Lucovnik M, Avcin T, Krivec U. Respiratory morbidity, lung function and fitness assessment after bronchopulmonary dysplasia. Journal of Perinatology 2015;35(12):1037-1042.

7. Korhonen PH, Suursalmi PH, Kopeli T, Nieminen R, Lehtimaki L, Luukkaala T, Korppi M, Saari A, Moilanen E, Tammela OKT. Inflammatory Activity at School Age in Very Low Birth Weight Bronchopulmonary Dysplasia Survivors. Pediatric Pulmonology 2015;50(7):683-690.

8. Beckmann J, Bennett K, Bolton C, Marlow N, Hurst J. Respiratory outcomes in young adults following extremely preterm birth. European Respiratory Journal Conference: European Respiratory Society Annual Congress 2016;48(Supplement 60).

9. Cazzato S, Ridolfi L, Bernardi F, Faldella G, Bertelli L. Lung Function Outcome at School Age in Very Low Birth Weight Children. Pediatric Pulmonology 2013;48(8):830-837.

10. Caskey S, Gough A, Rowan S, Gillespie S, Clarke J, Riley M, Megarry J, Nicholls P, Patterson C, Halliday HL, Shields MD, McGarvey L. Structural and Functional Lung Impairment in Adult Survivors of Bronchopulmonary Dysplasia. Annals of the American Thoracic Society 2016;13(8):1262-1270.

11. Kaplan E, Bar-Yishay E, Prais D, Klinger G, Mei-Zahav M, Mussaffi H, Steuer G, Hananya S, Matyashuk Y, Gabarra N, Sirota L, Blau H. Encouraging pulmonary outcome for surviving, neurologically intact, extremely premature infants in the postsurfactant era. Chest 2012;142(3):725-733.

12. Vollsaeter M, Clemm HH, Satrell E, Eide GE, Roksund OD, Markestad T, Halvorsen T. Adult respiratory outcomes of extreme preterm birth a regional cohort study. Annals of the American Thoracic Society 2015;12(3):313-322.

13. Mieskonen ST, Malmberg LP, Kari MA, Pelkonen AS, Turpeinen MT, Hallman NMK, Sovijarvi ARA. Exhaled nitric oxide at school age in prematurely born infants with neonatal chronic lung disease. Pediatric Pulmonology 2002;33(5):347-355.

14. Hafkamp-de Groen E, Sonnenschein-van Der Voort AMM, Mackenbach JP, Duijts L, Jaddoe VWV, Moll HA, Hofman A, De Jongste JC, Raat H. Socioeconomic and sociodemographic factors associated with asthma related outcomes in early childhood: The generation R study. PLoS ONE 2013;8(11).

15. Holster IL, Sonnenschein-van der Voort AMM, Duijts L, De Jongste JC, Jaddoe VW, Perez-Perez GI, Blaser MJ, Moll HA, Kuipers EJ. Maternal helicobacter pylori colonization is not associated with asthma symptoms, airway inflammation and airway resistance in their children until the age of 6 years: The generation r study. Gastroenterology 2013;1):S5.

16. Rucci E, den Dekker HT, de Jongste JC, Steenweg-de-Graaff J, Gaillard R, Pasmans SG, Hofman A, Tiemeier H, Jaddoe VWV, Duijts L. Maternal fatty acid levels during pregnancy, childhood lung function and atopic diseases. The Generation R Study. Clinical and Experimental Allergy 2016;46(3):461-471.

17. Pike KC, Inskip HM, Robinson SM, Cooper C, Godfrey KM, Roberts G, Lucas JS. The relationship between maternal adiposity and infant weight gain, and childhood wheeze and atopy. Thorax 2013;68(4):372-379.

18. Vollsaeter M, Skromme K, Satrell E, Clemm H, Roksund O, Oymar K, Markestad T, Halvorsen T. Children Born Preterm at the Turn of the Millennium Had Better Lung Function Than Children Born Similarly Preterm in the Early 1990s. PLoS ONE [Electronic Resource] 2015;10(12):e0144243.

19. Malmberg LP, Pelkonen AS, Malmstrom K, Saarinen KM, Kajosaari M, Hakulinen A, Makela MJ. Very low birth weight and respiratory outcome: association between airway inflammation and hyperresponsiveness. Ann Allergy Asthma Immunol 2013;111(2):96-101.
